# Localization of Human RNase Z Isoforms: Dual Nuclear/Mitochondrial Targeting of the *ELAC2* Gene Product by Alternative Translation Initiation

**DOI:** 10.1371/journal.pone.0019152

**Published:** 2011-04-29

**Authors:** Walter Rossmanith

**Affiliations:** Center for Anatomy and Cell Biology, Medical University of Vienna, Vienna, Austria; Newcastle University, United Kingdom

## Abstract

RNase Z is an endonuclease responsible for the removal of 3′ extensions from tRNA precursors, an essential step in tRNA biogenesis. Human cells contain a long form (RNase Z^L^) encoded by *ELAC2*, and a short form (RNase Z^S^; *ELAC1*). We studied their subcellular localization by expression of proteins fused to green fluorescent protein. RNase Z^S^ was found in the cytosol, whereas RNase Z^L^ localized to the nucleus and mitochondria. We show that alternative translation initiation is responsible for the dual targeting of RNase Z^L^. Due to the unfavorable context of the first AUG of *ELAC2*, translation apparently also starts from the second AUG, whereby the mitochondrial targeting sequence is lost and the protein is instead routed to the nucleus. Our data suggest that RNase Z^L^ is the enzyme involved in both, nuclear and mitochondrial tRNA 3′ end maturation.

## Introduction

tRNAs are generally synthesized as immature precursors with extensions on both ends [1]. Removal of these extra sequences is essential for tRNA function. Extraneous nucleotides at the 5′ end are removed by a single endonucleolytic cleavage catalyzed by an enzyme known as RNase P [2], [3]. 3′ end processing is either accomplished by precise endonucleolytic cleavage too, or by the concerted action of multiple exo- and endonucleases [1], [2], [4]. The enzyme responsible for the first, exclusively endonucleolytic pathway is called RNase Z (or tRNase Z; EC 3.1.26.11) [2], [5]–[8].

RNase Z enzymes belong to the metallo-β-lactamase superfamily. In addition to the diagnostic α–β/β–α β-lactamase-fold, a protruding flexible arm involved in binding tRNA precursors is characteristic for RNase Z structures. There are two forms of RNase Z, one of 280 to 360 amino acids (RNase Z^S^) found in archaea and many, but not all bacteria and eukarya, and one, more than twice this length (750–930 amino acids; RNase Z^L^), exclusively found in eukarya. The primary structure of RNase Z^L^ suggests that it has evolved from RNase Z^S^ by duplication. While all eukarya appear to have at least one RNase Z^L^ gene, some also have RNase Z^S^, or more than one gene encoding a long and/or short form of RNase Z.

In the human genome RNase Z^S^ is encoded by *ELAC1* and RNase Z^L^ by *ELAC2* (the gene names refer to their homology to the *Escherichia coli* RNase Z gene *elaC*). Recombinant proteins derived from both genes have RNase Z activity, i.e., cleave tRNA precursors at their 3′ end *in vitro* [9]. The predicted mitochondrial targeting sequence of *ELAC2* suggests that RNase Z^L^ localizes to mitochondria, whereas RNase Z^S^ might accordingly be assumed to be responsible for the processing of nuclear encoded tRNAs. However, due to a lack of systematic approaches, the available experimental evidence is largely inconclusive. RNase Z^S^ was found mainly in the cytosol upon biochemical fractionation [10] and subcellular localization of RNase Z^L^ (ELAC2) was either reported to be mostly cytosolic [11], exclusively mitochondrial [12], or ubiquitous (cytosol, nucleus, and mitochondria) [13].

To clarify the subcellular distribution of the two human RNase Z isoforms we studied the localization of enhanced green fluorescent protein (EGFP)-tagged variants in human cell lines. By bioinformatics and experimental manipulation of the translation initiation site of RNase Z^L^ (ELAC2) we moreover revealed the basic mechanism underlying the observed dual nuclear/mitochondrial targeting of RNase Z^L^.

## Results

### Subcellular localization of EGFP-tagged RNase Z^L^ and RNase Z^S^

Different bioinformatic tools invariably predicted human RNase Z^L^ to be targeted to mitochondria by its N-terminal amino acid sequence. Moreover, amino acids 28 to 35 are predicted to comprise a nuclear localization signal. In contrast, RNase Z^S^ appears to neither contain a mitochondrial targeting sequence nor a nuclear localization signal.

**Figure 1:**
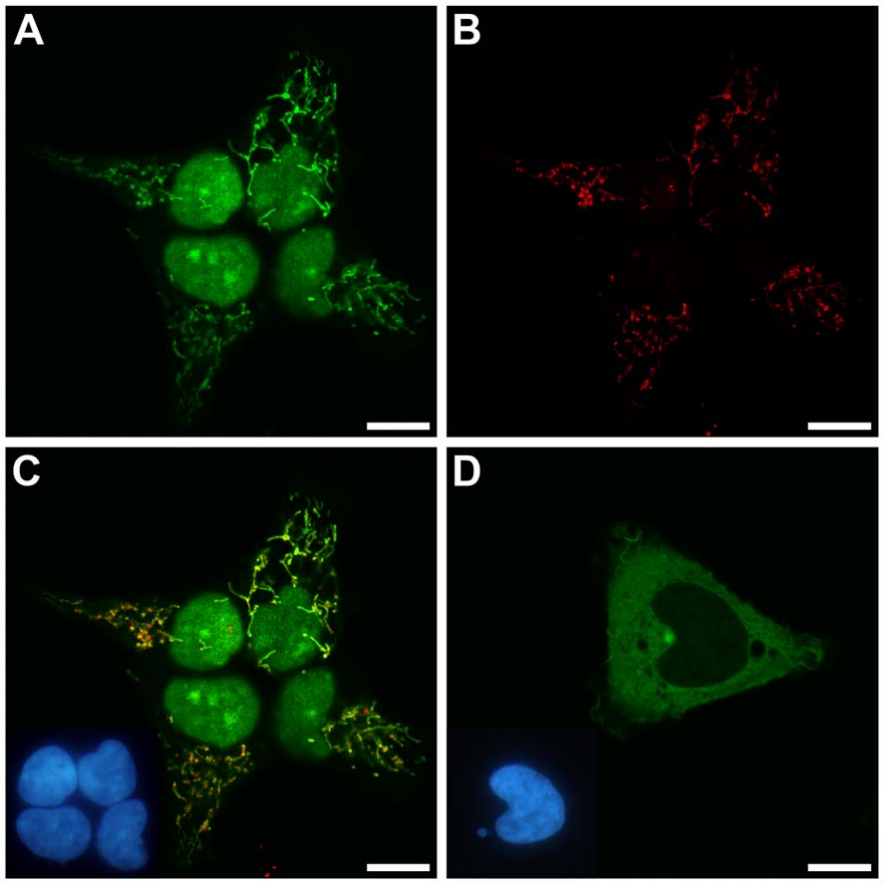
Subcellular localization of EGFP-tagged RNase Z^L^ and RNase Z^S^ in 293 cells. (A) RNase Z^L^-EGFP; (B) immunofluorescence localization of subunit I of cytochrome *c* oxidase (mitochondrial staining) in the cells shown in (A); (C) overlay of (A) and (B) with nuclei shown as inset; (D) RNase Z^S^-EGFP with nuclei shown as inset. Confocal laser scanning microscopy (scale bar, 10 µm); insets showing nuclei by epifluorescent microscopy.

To address the localization of both proteins experimentally we expressed their complete coding sequences tagged by EGFP in human 293 cells. To avoid interfering with putative N-terminal sorting signals we fused the EGFP-tag to the C-termini of the proteins. RNase Z^L^ localized to the nucleus and the mitochondria of transfected cells, but showed no cytosolic fluorescence (Figure 1A–C). RNase Z^S^ on the other hand, was found primarily in the cytosol (Figure 1D). Still, RNase Z^S^-EGFP expressing cells also displayed some weak nuclear fluorescence. In contrast, EGFP alone distributed evenly throughout the cytosol and the nucleus (data not shown).

### Dual nuclear/mitochondrial targeting of RNase Z^L^ by alternative translation initiation

**Figure 2:**
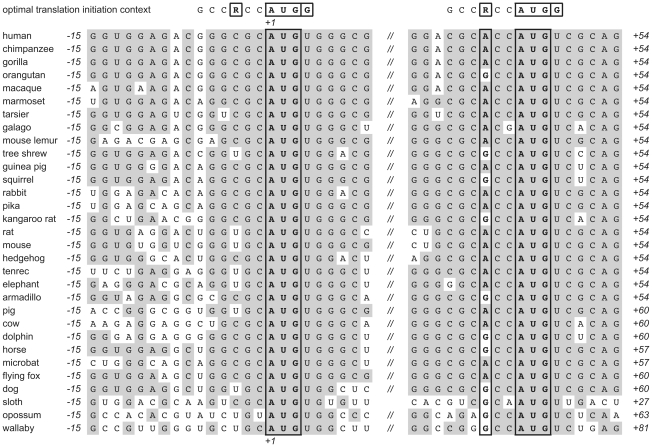
Alignment of the two putative initiation sites of mammalian RNase Z^L^ mRNAs (*ELAC2* homologs). Optimal (consensus) translation initiation context of mammalian mRNAs [14] shown on top. R^−3^, AUG^Start^, and G^+4^ outlined; shading indicates identity to more than 50% of the sequences.

How does the nuclear fraction of RNase Z^L^ escape the mitochondrial import pathway? The assigned start codon of human RNase Z^L^ (*ELAC2*) appears to be in a suboptimal configuration for efficient initiation of translation (Figure 2). Initiation sites in mammalian mRNAs usually conform at least in part to the sequence GCCRCCAUGG [14]. The strongest determinants for translation initiation besides the AUG itself are a purine (R), preferably an A, at −3 and, especially in the absence of A^−3^, a G in position +4. The first AUG of human RNase Z^L^ mRNA lacks both, A^−3^ and G^+4^. The next AUG, 45 nucleotides downstream, is preceded by ACC and thus in a much more favorable context for translation initiation. However, proteins translated from this second putative initiation site would lack 15 N-terminal amino acids and thereby most of the predicted mitochondrial targeting sequence. In fact, unlike full length RNase Z^L^ (826 amino acids), the slightly shortened RNase Z^L^ (811 amino acids) is no longer predicted to localize to mitochondria by any of the employed bioinformatic tools, whereas the predicted nuclear localization signal is not affected. The peculiar configuration of the two putative start codons is conserved among mammalian *ELAC2* homologs (Figure 2). While position −3 is never occupied by a purine in the case of the first AUG, the second AUG is always preceded by a purine at −3. Also other, less important determinants for efficient translation initiation (C^−4^ and C^−2^) conform more closely to the consensus sequence in the case of the second putative initiation site. The second AUG thus invariably appears to be in a better initiation context than the first.

**Figure 3:**
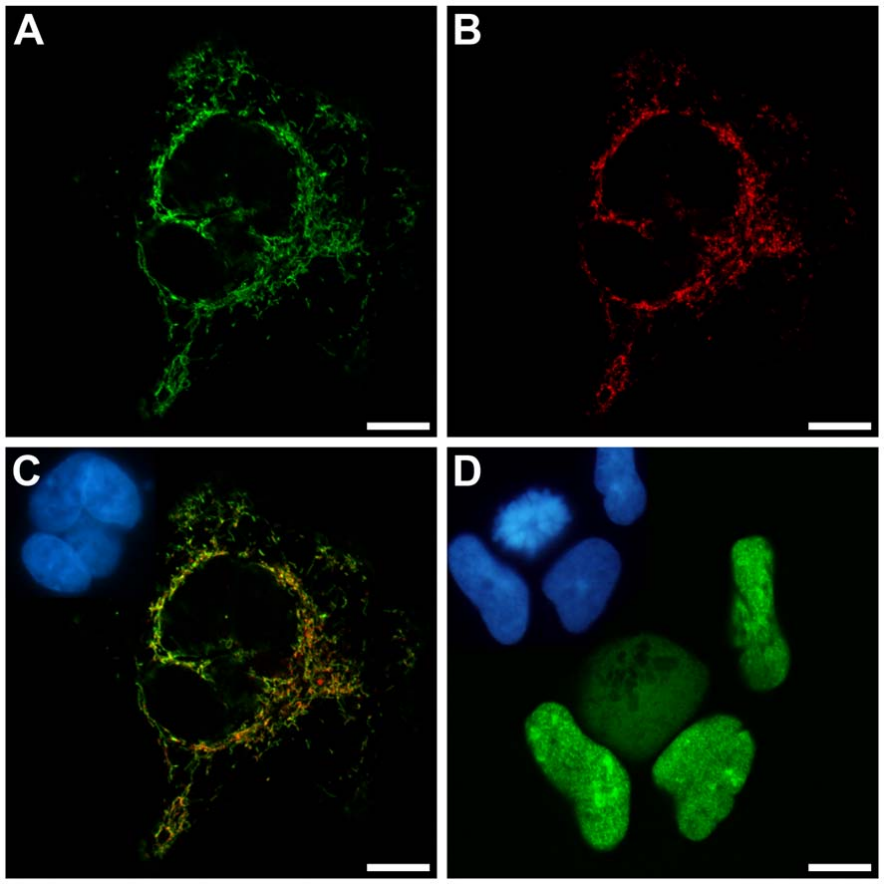
Subcellular localization of EGFP-tagged RNase Z^L^ variants with modified translation initiation sites in 293 cells. (A) RNase Z^L^-EGFP variant with an optimized translation initiation context of the first AUG; (B) immunofluorescence localization of subunit I of cytochrome *c* oxidase (mitochondrial staining) in the cells shown in (A); (C) overlay of (A) and (B) with nuclei shown as inset; (D) RNase Z^L^-EGFP variant without the first 15 amino acids, nuclei shown as inset. Confocal laser scanning microscopy (scale bar, 10 µm); insets showing nuclei by epifluorescent microscopy.

To test whether the observed dual nuclear/mitochondrial localization of human RNase Z^L^ is indeed due to alternative translation initiation we prepared two variants of the *ELAC2* cDNA and analyzed their subcellular localization. On the one hand, we changed the sequence preceding the first AUG to the more favorable initiation context GTCACC, on the other hand, we deleted the first 15 amino acids to allow translation from the second AUG only. Modification of the nucleotides preceding the first AUG to conform to the consensus gave rise to an RNase Z^L^ exclusively localized to mitochondria (Figure 3A–C). Others also recently reported a mitochondria-restricted localization of RNase Z^L^; notably, their epitope-tagged RNase Z^L^ was expressed from an (unintentionally) initiation context-optimized cDNA too [12]. As further predicted by our model, a cDNA devoid of the first few codons resulted in an RNase Z^L^ variant exclusively found in the nucleus (Figure 3D; with the expected exception of cells undergoing mitosis).

In contrast to previous reports [11], [13] we did not observe any appreciable cytosolic RNase Z^L^ in any of our experiments with the exception of cells currently undergoing mitosis. Apparently, nuclear RNase Z^L^ was also completely re-imported after re-formation of the nuclear membrane. The localization pattern of RNase Z^S^, RNase Z^L^, and the two RNase Z^L^ translation initiation variants was identical in 143B osteosarcoma cells (Figure S1).

## Discussion

Determining an enzyme's subcellular localization is crucial to fully comprehend its biology. Eukaryal cells generally transcribe and process tRNA precursors in the nucleus and mitochondria (plants moreover in chloroplasts). All eukarya also have one RNase Z^L^ gene, and it appears evolutionary optional to have further RNase Z genes encoding either short or additional long forms. Consistently, RNase Z^L^ is targeted to the nuclear and the mitochondrial compartment in organisms where it is the only form of RNase Z, like *Saccharomyces cerevisiae* or *Drosophila melanogaster* [15], [16]. Yet, despite the additional presence of an RNase Z^S^, we show that human RNase Z^L^ localizes to nuclei and mitochondria too, while RNase Z^S^ is found mainly in the cytosol. Only if there is more than one RNase Z^L^ gene, the general dual nuclear/mitochondrial localization of RNase Z^L^ appears dispensable. In *Arabidopsis thaliana* one of the two RNases Z^L^ is still dual targeted while the other one is found in mitochondria only [17]. In the yeast *Schizosaccharomyces pombe* finally, the two RNases Z^L^ are confined to the nucleus and the mitochondria, respectively [18]. Interestingly, also in plants, where there are up to four RNases Z, only the long forms localize to nuclei and/or mitochondria, whereas the two RNases Z^S^ are found in the cytosol and in chloroplasts, respectively [17].

The cytosolic localization of human RNase Z^S^ indicates that it is not involved in the processing of tRNA primary transcripts. This conclusion is consistent with its low tRNA processing efficiency *in vitro* [19] and with the fact that its gene (*ELAC1*) is part of a homozygous deletion in a lung cancer cell line, and thus apparently dispensable for cell survival and growth [20]. Unfortunately, there are currently no more clues to the biological role of human RNase Z^S^.

In contrast, the subcellular localization of human RNase Z^L^ suggests a role in the 3′ end maturation of nuclear and mitochondrial encoded tRNAs. Such a dual role has in fact been demonstrated for *D. melanogaster* RNase Z^L^ [16]. It is unclear however, whether RNase Z^L^ is generally the only nuclease responsible for tRNA 3′ end formation. While there is evidence for alternative, exonucleolytic pathways for nuclear tRNA maturation in different eukarya [1], the polycistronic nature of mitochondrial tRNA precursors appears to strictly require an endonuclease [21]. Notably, only mitochondrial and chloroplast RNases Z are essential in *A. thaliana* [17]. However, because both RNases Z^L^ are targeted to mitochondria, only the double mutant is lethal in this case [17].

The basic mechanism underlying the dual nuclear/mitochondrial localization of human RNase Z^L^ appears to be the alternative use of two translation initiation sites. The first AUG of mammalian RNase Z^L^ is in a poor context for initiation and thus apparently passed by most scanning ribosomes, while the second AUG more closely conforms to a consensus initiation site and appears to be used preferentially. This mechanism of alternative translation initiation has been described as context-dependent leaky scanning [14]. In the case of mammalian RNase Z^L^ downstream initiation results in the loss of most of the mitochondrial targeting signal, whereby the protein translated from the second AUG is no longer imported into the mitochondrial matrix, but routed to the nucleus via its nuclear localization signal. Accordingly, experimental optimization of the context of the first AUG resulted in an RNase Z^L^ exclusively targeted to mitochondria, whereas its elimination produced nuclear RNase Z^L^ only. This moreover implies that the mitochondrial targeting signal is either dominant over the nuclear localization signal, or the latter masked by the presence of the former [22]. A similar localization mechanism has been proposed for two other human nuclear/mitochondrial proteins, DNA ligase III and DNA topoisomerase IIIα [23], [24].

Context-dependent leaky scanning does not exclude that there are other, currently unknown mechanisms involved in regulating the balance between nuclear and mitochondrial RNase Z^L^. We previously reported a reduction in mitochondrial RNase Z activity in human cells lacking mitochondrial DNA (ρ^0^ cells) [25]. More recently, it was reported that the RNase Z^L^ coding *ELAC2* mRNA is in fact down-regulated in ρ^0^ cells [12]. It is not known whether this down-regulation is accompanied by the preferential targeting of the remaining RNase Z^L^ to the nucleus or affects the cellular RNase Z^L^ pool as a whole and thereby possibly contributes to the slower growth of ρ^0^ cells.

We did not observe any RNase Z^L^ in the cytosol. Previously, others had reported a predominantly cytosolic localization of ELAC2 upon biochemical fractionation [11] and a ubiquitous cellular localization of RNase Z^L^ by immunofluorescence [13]. Experimental design and methodological approach appear to account for these discrepancies. A protein tagged at its N-terminus by two FLAG epitopes was expressed from an optimized initiation site in the first study [11]. Thereby the N-terminal mitochondrial targeting signal was displaced and initiation from the downstream AUG, responsible for translation of nuclear RNase Z^L^, prevented. The N-terminal tag might have additionally masked the nuclear localization signal [22]. Some nuclear proteins moreover have a strong tendency to leak to the cytosol upon biochemical fractionation. Concerning the latter study [13], the uniform immunofluorescence throughout the cell casts doubts on the specificity of the antibody used. In the end, it is unlikely that the EGFP attached to the C-terminus of RNase Z^L^ in our study prevented its cytosolic localization, as this was neither the case with EGFP alone nor with EGFP tagged RNase Z^S^.

### Concluding remarks

Animal cells use two radically different enzymes for the processing of tRNA 5′ ends in the nucleus and mitochondria [26]–[28]. In contrast, key enzymatic components appear to be shared for the formation of tRNA 3′ ends. RNase Z^L^ is localized to both compartments of tRNA biogenesis, and considering that only a single gene (*TRNT1*) for ATP(CTP):tRNA nucleotidyltransferase, the CCA-adding enzyme has been identified in the human genome, it appears reasonable to assume it is shared as well [29]. An analysis of TRNT1's N-terminus and putative translation initiation sites at least suggests that it employs the same basic mechanism for dual localization as RNase Z^L^ (unpublished analysis).

## Materials and Methods

### Bioinformatics

For the prediction of subcellular localization we used the following tools: LOCtree [30], TargetP [31], MitoProt II [32], Predotar [33], and PredictNLS [34].

Mammalian RNase Z^L^ gene sequences were retrieved from Ensembl [35]. Sequence alignments were generated with MacVector 6.5 (Oxford Molecular).

### cDNA expression plasmids

The coding sequences of *ELAC1* (NM_018696) and *ELAC2* (NM_018127) including 15 nucleotides of 5′ untranslated region preceding the start codon were PCR amplified from HeLa cell (ATCC# CCL-2) cDNA using primers containing *Bam*HI and in-frame *Xba*I restriction sites. PCR products were cloned into a pcDNA4/TO (Invitrogen) that was modified by insertion of the EGFP coding sequence from pEGFP-N3 (Invitrogen) into its *Apa*I site. Proteins expressed from these plasmids are composed of their complete native amino acid sequence fused at their C-terminus via a short linker peptide (SerArgGlyProAlaThr) to EGFP.

The native *ELAC2* expression plasmid was modified by site-directed mutagenesis using the QuikChange (Stratagene) protocol: GTCACC replaced (i) the natural 5′ untranslated region, or (ii) the 5′ untranslated region plus the first 45 nucleotides of coding sequence.

### Cell lines, staining, and microscopy

Stable T-REx-293 (Invitrogen) cell lines were generated as described recently [28]. For each expression cassette we isolated and analyzed several clones.

Cells were grown on cover slips and expression induced with tetracycline at 0.2 µg/ml. Paraformaldehyde fixed cells were stained with bisbenzimid H33342 and embedded in Kaiser's glycerol gelatin. For mitochondrial staining cells were fixed, permeabilized with 0.1% Triton X-100, and incubated with a monoclonal antibody specific for subunit I of cytochrome *c* oxidase (Molecular Probes A6403) at 5 ng/µl. For detection biotinylated anti mouse IgG and Texas Red conjugated streptavidin were used. A conventional epifluorescent and a dual-channel confocal laser scanning microscope (Olympus FluoView) were used. Pictures were processed with the FluoView application software (Olympus).

A 143B (ATCC# CRL-8303) derived cell line stably expressing DsRed2 (Clontech) targeted to mitochondria by the signal peptide of cytochrome *c* oxidase subunit VIII was selected. This cell line was transfected with the *ELAC* expression plasmids by a magnet assisted transfection procedure according to the instructions of the supplier (IBA BioTAGnology).

## Supporting Information

Figure S1 **Subcellular localization of EGFP-tagged RNase Z^S^, RNase Z^L^, and RNase Z^L^ variants with modified translation initiation sites in 143B cells.** (A) RNase Z^S^-EGFP; (B) RNase Z^L^-EGFP with native translation initiation context; (C) RNase Z^L^-EGFP variant with an optimized translation initiation context of the first AUG; (D) RNase Z^L^-EGFP variant without the first 15 amino acids; (E–H) DsRed2 labeled mitochondria of cells shown in the same row; (I–L) nuclear staining of cells shown in the same row.
(TIF)
