# Supplementary figures and images for: Localization of Human RNase Z Isoforms: Dual Nuclear/Mitochondrial Targeting of the ELAC2 Gene Product by Alternative Translation Initiation
Source: PLoS One. 2011 Apr 29;6(4):e19152. doi: 10.1371/journal.pone.0019152 (PMC3084753; doi:10.1371/journal.pone.0019152)

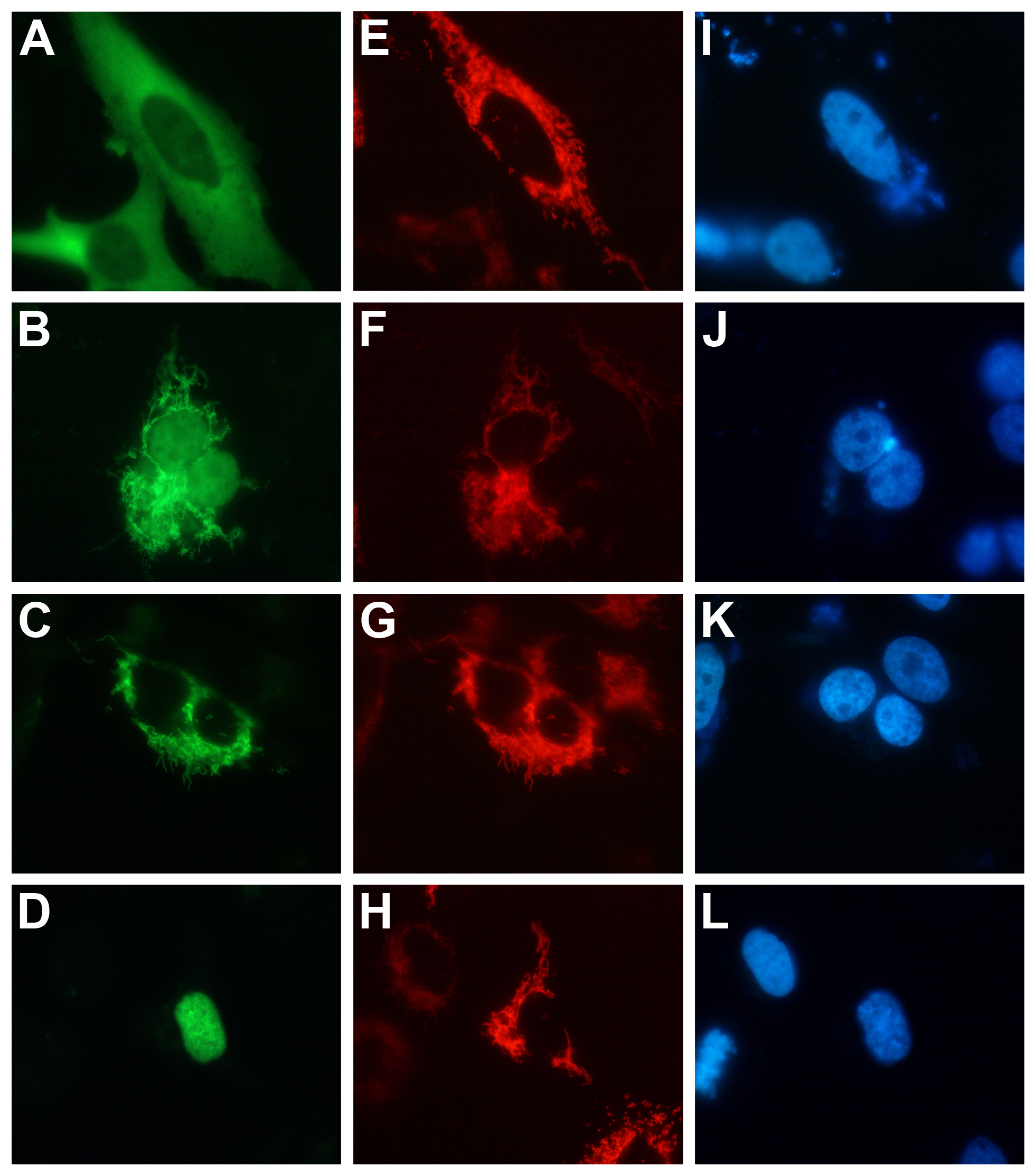

Supplement: Figure S1 — Subcellular localization of EGFP-tagged RNase ZS, RNase ZL, and RNase ZL variants with modified translation initiation sites in 143B cells. (A) RNase ZS-EGFP; (B) RNase ZL-EGFP with native translation initiation context; (C) RNase ZL-EGFP variant with an optimized translation initiation context of the first AUG; (D) RNase ZL-EGFP variant without the first 15 amino acids; (E–H) DsRed2 labeled mitochondria of cells shown in the same row; (I–L) nuclear staining of cells shown in the same row. (TIF) [file pone.0019152.s001.tif]
